# 

**Published:** 1890-07

**Authors:** 


### CONTENTS—JULY, 1890.—VOL. VI., NO. 1.

ORIGINAL ARTICLES—

The Limitations of the Healing Art. D. R. Wallace, A. M., M. D., LL. D . . . . . . . . . . . . . . . . . . . . . . . . . . . . 1

Dysentery. A. Garwood, M. D . . . . . . . . . . . . . . . . . . . . . . 13

" Discussion before the Austin District Medical Society . . . . . . . . . . . . . . . . . . . . . . . . . . . . . . . . . . . 18

Gastro-Enterostomy. Seth M. Morris—Student . . . . . . 22

EDITORIAL—

Doctors and "Doctors" . . . . . . . . . . . . . . . . . . . . . . . . . . . 27

Texas State Medical Association—Delinquent Members 33

Dr. R. H. L. Bibb . . . . . . . . . . . . . . . . . . . . . . . . . . . . . . . . 34

MEDICAL NEWS AND MISCELLANY—

Deaths, Removals, Marriages, Society Notes, Therapeutic Suggestions, etc., thirty paragraphs of news . . 35

BOOK NOTICES—

Orthopœdic Surgery; Wm. Wood & Co.; Keating's Cyclopedia, Vol. III.—Lippincott. . . . . . . . . . . . . . . . . 41

PUBLISHER'S NOTICES—

Or Therapeutic Hints . . . . . . . . . . . . . . . . . . . . . . . . . . . . . 44

